# Minimum inhibitory concentrations and resistance for selected antimicrobial agents (including imipenem, linezolid and tigecycline) of bacteria obtained from eye infections

**Published:** 2020

**Authors:** Virgilio Galvis, Alejandro Tello, Walter Sánchez, Paul Camacho, Donaldo Villarreal, Diana García

**Affiliations:** * Centro Oftalmológico Virgilio Galvis, Floridablanca, Colombia; ** Fundación Oftalmológica de Santander FOSCAL, Floridablanca, Colombia; *** Universidad Autónoma de Bucaramanga UNAB, Bucaramanga, Colombia; **** Laboratorio Clínico Higuera Escalante, Ocular Microbiology, Floridablanca, Colombia

**Keywords:** minimum inhibitory concentration, antimicrobial resistance, Etest, infectious keratitis, endophthalmitis

## Abstract

**Objective:** To determine bacteria obtained from eye infections, both resistance and minimal inhibitory concentration (MIC) to gatifloxacin, moxifloxacin, tigecycline, linezolid and imipenem, in vitro.

**Methods:** A cross-sectional descriptive study was undergone with 50 samples from 50 eyes of patients diagnosed with keratitis or endophthalmitis, who came to a consultation at the Fundación Oftalmológica de Santander (Floridablanca, Colombia) from August to November 2014. The MICs of the isolated microorganisms were established through Etest® strips (BioMérieux SA, Marcy-l'Etoile - France).

**Results:** Of the 50 samples in total, 17 different bacteria species or groups were isolated. The main isolate for gram-positives was *Methicillin Resistant Coagulase-Negative Staphylococcus* (17 samples), and for gram-negatives was *Pseudomonas aeruginosa* (6 samples). The susceptibility percentages sorted from highest to lowest for gram-positive isolates (n=38) were: imipenem 90.3%, linezolid 87.9%, tigecycline 78.1%, gatifloxacin 68.8% and moxifloxacin 68.8%. For gram-negative isolates (n=12), they were: imipenem 72.7%, gatifloxacin 70%, moxifloxacin 66.7% (no reference cut-off points were found for *Pseudomonas aeruginosa*), tigecycline 22.2%, and linezolid 0% (as expected according to its inhibition spectrum).

**Conclusions:** Although fourth generation fluoroquinolones are currently the preferred initial empirical monotherapy in our practice, given the increasing bacterial resistance, in cases in which gram-positive bacteria were isolated in the initial staining imipenem, linezolid or tigecycline could be used as an alternative. On the other hand, for cases of gram-negative bacteria, no antimicrobial susceptibility exceeded 80%, so using two antimicrobials looking for a synergy between them could be a better option.

**Abbreviations:** S = Susceptibility, IS = Intermediate susceptibility, R = Resistance

## Introduction

One of the key advances in modern medicine was the introduction of antibiotics in the clinical context, but it was rapidly found that microorganisms could become resistant to them, so there is an increasing need of studying new antibiotic molecules and combinations [**1**].

Some of the likely causes of increasing antimicrobial resistance (AMR) include excessive and occasionally unneeded prescription of antibiotics, over-the-counter acquisition, poor hygiene and sanitation, inappropriate or incomplete treatments, among others. To slow the AMR and avert the propagation of resistant germs, campaigns of rational antimicrobial use and self-medication avoidance have been fostered [**1**,**2**].

Bacterial ophthalmological infections such as microbial keratitis (due to the fact that it is often accompanied by loss of the epithelium, is also known with the most nonspecific term “corneal ulcer”) and endophthalmitis can lead to serious visual sequelae, hence the importance of knowing which antibiotics are effective for their treatment. However, the process of choosing the most adequate antibiotic for a determined case is based on different variables including availability, costs, dosage, adverse effects, patient adherence to treatment, but especially good tissue penetration, and low Minimum Inhibitory Concentration (MIC). The interpretation of the latter factor in terms of susceptibility has to be based on systemic parameters that may not correlate with the conditions of topical treatment, which does not have specific standards but its parameters would probably increase due to high antibiotic tissue levels achieved by topical antibiotic application. Nevertheless, the susceptibility interpretation based on serum standards serves as a guide for analyzing the bacteria’s state of resistance in our context, in order to obtain good clinical outcomes when treating patients [**2**-**4**].

**Fig. 1:**
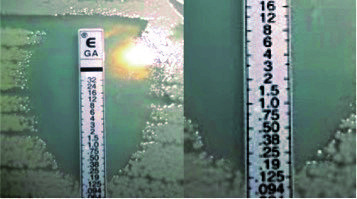
Etest® with gatifloxacin showing an ellipse of inhibition of microbial growth intersecting the scale on the strip at the point of 0.125 µg/ ml, representing that this value is the MIC of the antibacterial substance for this germ

The MIC is defined as the lowermost concentration of a chemical that impedes observable growth of a microorganism after incubating the media during 16-20 hours, extending over a night (this period is longer for anaerobes, which require more incubation time for their growth). Typically, the MIC of an antibacterial drug is established with a complex technique that includes a series of dilutions of the substance (in agar or in culture broth) in which microorganisms are inoculated and growth is evaluated. Nowadays, there are also automated systems that determine it. Additionally, other commercial methods are available, including Etest® (Epsilometer test) strips, that do not require high technology, and simplify that process. The Etest® comprises a strip of a plastic, which contains a predefined gradient of antibiotic concentrations that, when applied to agar plates where the microorganisms were inoculated, and after incubation, permits observing ellipses of microbial inhibition (**Fig. 1**). The MIC value is given by the place where the inhibition ellipse intersects with the strip, where a scale of concentrations is printed on [**5**,**6**].

This approach has been employed to determine MIC in numerous studies including isolates from human ophthalmic infections [**4**,**7**].

Using this system, we determined the MIC of various antibiotics for a variety of bacteria isolated from cases of infectious keratitis and endophthalmitis. We investigated quinolones of fourth generation, which are presently used topically for corneal infections (gatifloxacin and moxifloxacin), and furthermore antibiotics that are not customarily employed in this context (i.e. imipenem, tigecycline and linezolid) and could potentially become alternate options for treatment.

## Methods

A cross-sectional study was performed with samples taken from cases with keratitis and endophthalmitis, with positive cultures for bacteria, at the Fundación Oftalmológica de Santander – FOSCAL (Floridablanca, Colombia) between August and November 2014. MICs in µg/ ml were established with Etest® semi-quantitative technique (BioMérieux SA, Marcy-l'Etoile - France) for: gatifloxacin, moxifloxacin, tigecycline, linezolid and imipenem. 

Bacterial keratitis specimens were obtained by ophthalmologists at the slit lamp. Endophthalmitis samples corresponded to vitreous humor and aqueous humor and were taken in the surgical room through vitrectomy via pars plana or through an anterior chamber paracentesis.

The specimens were implanted in an enriched broth and then reinoculated in agar. The Etest® strips were positioned to establish the minimal inhibitory concentrations and the reading was done by an ocular microbiologist.

The MIC50 for each bacterium was computed as explained by Kowalski and co-authors: the MIC50 was deemed as the concentration of antimicrobial that inhibited the growth of half (i.e. 50%) of the isolates evaluated. For example, if we assume that the MICs of 10 isolates were found to be ordered from minimum to maximum, 2, 2, 4, 4, 8, 16, 16, 16, 32, and 32 µg/ ml, the MIC50 would be the value in the place n*0.5. Therefore, MIC50 for this series of 10 is the MIC on the 5th position, i.e. 8 µg/ ml. In an even number of values like this one, MIC50 will not be equivalent to the median, because the latter is calculated by averaging the two central values (in this example it would be 12 µg/ ml). On the other hand, MIC50 in an odd series of MIC values, corresponds to the position (n+1)*0.5 and will be equivalent to the median. For example, in the series of 11 MIC values: 2, 2, 2, 4, 4, 4, 8, 8, 8, 16, and 32 µg/ ml, MIC50 will be MIC at the position (11+1)*0.5 = 6 (after sorting the values in ascending order). Therefore, MIC50 will be 4 µg/ ml, which also corresponds to the median of this series [**4**].

The correspondence among the MIC and the susceptibility, intermediate susceptibility or resistance of the germ was established following the guidance of the Clinical & Laboratory Standards Institute of the United States (acronym: CLSI). When no standards indicated by this guide were located for a given microorganism, other sources were sought (European Committee on Antimicrobial Susceptibility Testing (EUCAST) and the U.S. Food and Drug Administration (FDA)) [**8**,**9**].

MIC values are discrete quantitative variables, which, unlike continuous variables, involve distinct or separate numbers, rather than an uncountable set of values in a range. In addition to MIC50, other central tendency and variability measures of MIC values (median, mode, minimum and maximum) can be used as descriptive coefficients for discrete data. Therefore, they were also calculated for each microorganism and antibiotic in this study [**4**].

## Results

50 samples from 50 eyes were included and distributed as it follows: 40 samples of bacterial keratitis, and 6 samples of vitreous humor and 4 samples of aqueous humor in cases of bacterial endophthalmitis. Of these samples, 17 dissimilar bacterial species in total (or groups when it was not achievable to establish the species) were isolated (**Table 1**).

**Table 1:** Percentage of different bacteria isolated from bacterial keratitis and endophthalmitis samples

Microorganism	% Isolated bacteria n=50 samples
Gram - Bacteria	Number of Isolates
*Escherichia Coli*	1
*Kingella denitrificans*	1
*Klebsiella enterobacter*	1
*Pseudomona stutzeri*	1
*Pseudomonas aeruginosa*	6
*Serratia marcescens*	1
*Sphingomonas paucimobilis*	1
Total of Gram -	12
Gram + Bacteria	
*Bacillus Cereus*	3
*Bacillus sp*	1
*Enterococcus durans*	1
*Globicatella sanguinis*	1
*Micrococcus luteus*	1
*Methicillin Resistant Staphylococcus aureus*	5
*Coagulase-Negative Staphylococcus*	5
*Methicillin Resistant Coagulase-Negative Staphylococcus*	17
*Methicillin Resistant Staphylococcus haemolyticus *	1
*Alpha hemolytic Streptococcus *	3
Total of Gram +	38

Of the 31 Gram-positive isolates, in which it was possible to establish the susceptibility as stated by the standards, 90.3% were susceptible to imipenem. Two cases of alpha-hemolytic Streptococcus and one case of methicillin resistant Staphylococcus haemolyticus exhibited resistance.

Of the 32 Gram-positive isolates, in which susceptibility could be established as stated by the standards, 68.8% were susceptible to gatifloxacin. Eight cases of Methicillin Resistant Coagulase-Negative Staphylococcus showed resistance and two showed intermediate susceptibility.

Of the 32 Gram-positive isolates, in which the susceptibility could be established as stated by the standards, 68.8% were susceptible to moxifloxacin, 12.5% had intermediate susceptibility and 18.8% were resistant. However, we did not locate official cut-off points to determine susceptibility or resistance in 6 (15.7%) of the 38 isolated microorganisms.

Of the 33 Gram-positive isolates, in which the susceptibility could be established as stated by the standards, 87.9% were susceptible to linezolid, and four cases showed resistance (12.1%): an alpha- hemolytic Streptococcus, a methicillin-resistant haemolyticus Staphylococcus, a Globicatella sanguinis and an Enterococcus durans.

Of the 32 Gram-positive isolates, in which the susceptibility could be established following the standards, 78.1% were susceptible to tigecycline, and 21.9% were resistant. However, we did not locate standards for cut-off points to establish susceptibility or resistance in 6 (15.8%) of the 38 isolated microorganisms.

Of the 11 Gram-negative isolates, in which the susceptibility could be established as stated by the standards, 72.7% were susceptible to imipenem, one case (9.1%) presented resistance (Kingella denitrificans) and two cases (18.2%) of Pseudomonas Aeruginosa showed intermediate susceptibility.

Of the 10 Gram-negative isolates, in which the susceptibility could be established as stated by the standards, 70% were susceptible to gatifloxacin, two (20%) Pseudomonas Aeruginosa were intermediately susceptible and one (10%) was resistant.

Of the 3 Gram-negative isolates, in which the susceptibility could be established as stated by the standards, 66.7% were susceptible to moxifloxacin. One case of Serratia Marcescens was resistant (33.3%). However, we did not locate standards to determine susceptibility or resistance in 9 (75%) of the 12 isolated microorganisms.

Of the 9 Gram-negative isolates, in which the susceptibility could be established as stated by the standards, no microorganisms susceptible to linezolid were found and 100% were resistant.

Of the 9 Gram-negative isolates, in which the susceptibility could be established as stated by the standards, 22.2% were susceptible to tigecycline.

The results for each bacterium (if there were more than two isolates) and antibiotic substance, including MIC50, median, mode, maximal and minimal MIC values, and also the percentages of susceptibility or resistance, are found in **Table 2**.

**Table 2:** Descriptive Statistics of minimal inhibitory concentrations (MICs) (µg/ ml) for bacterial keratitis & endophthalmitis Isolates to 5 selected antibiotics

Antibiotic	n	Med.	Mode	MIC50	Min. MIC	Max. MIC	% S	% IS	% R
					Methicillin Resistant Coagulase-Negative *Staphylococcus*				
Imipenem	17	0.38	0.064	0.38	0.032	4.0	100	0	0
Moxifloxacin	17	0.094	0.094	1.0	0.094	8.0	47.1	23.5	29.4
Gatifloxacin	17	0.125	0.125	1.5	0.094	8.0	41.2	11.8	47.1
Linezolid	17	2.0	2.0	2.0	0.75	4.0	100	0	0
Tigecycline	17	0.125	0.125	0.25	0.047	4.0	88.2	0	11.8
					*Pseudomonas aeruginosa*				
Imipenem	6	3	4	3	1.5	4	66.7	33.3	0
Moxifloxacin	6	3	4	4	1.5	>32	nd	nd	nd
Gatifloxacin	6	2	4	2	0.75	>32	50	33.3	16.7
Linezolid	6	>256	>256	>256	>256	>256	0	0	100
Tigecycline	6	>256	>256	>256	>256	>256	0	0	100
					Coagulase-Negative *Staphylococcus*				
Imipenem	5	0.064	0.064	0.06	0.012	0.125	100	0	0
Moxifloxacin	5	0.094	0.125	0.09	0.032	0.125	100	0	0
Gatifloxacin	5	0.064	0.064	0.125	0.064	0.25	100	0	0
Linezolid	5	4	4	2	0.15	4	100	0	0
Tigecycline	5	0.064	N/A	0.094	0.032	1	80	0	20
					Methicillin resistant *Staphylococcus Aureus*				
Imipenem	4	0.127	0.064	0.19	0.064	0.5	100	0	0
Moxifloxacin	4	0.139	0.047	0.23	0.047	0.25	100	0	0
Gatifloxacin	4	0.253	0.38	0.38	0.125	0.38	100	0	0
Linezolid	4	1.75	1.5	2	1.5	4	100	0	0
Tigecycline	4	0.25	0.25	0.25	0.19	0.75	80	0	20
					Alpha hemolytic *Streptococcus*				
Imipenem	3	3	3	3.0	0.064	3.0	33.3	0	66.6
Moxifloxacin	3	0.25	N/A	0.25	0.016	0.75	nd	nd	nd
Gatifloxacin	3	0.38	N/A	0.38	0.125	1.5	100	0	0
Linezolid	3	1.25	N/A	1.25	0.5	>256	66.6	0	33.3
Tigecycline	3	0.064	0.064	0.064	0.064	0.38	66.6	0	33.3
					*Bacillus Cereus*				
Imipenem	3	0.094	N/A	0.094	0.047	0.125	nd	nd	nd
Moxifloxacin	3	0.125	N/A	0.125	0.064	0.19	nd	nd	nd
Gatifloxacin	3	0.125	N/A	0.125	0.064	0.19	nd	nd	nd
Linezolid	3	3	N/A	3	1	4	nd	nd	nd
Tigecycline	3	0.125	N/A	0.125	0.047	0.75	nd	nd	nd

In addition, we separately analyzed the cases corresponding only to bacterial keratitis (**Table 3**).

**Table 3:** Descriptive Statistics of minimal inhibitory concentrations (MICs) (µg/ ml) for bacterial keratitis Isolates to 5 selected antibiotics

Antibiotic	n	Med.	Mode	MIC50	Min. MIC	Max. MIC	% S	% IS	% R
					Methicillin Resistant Coagulase-Negative *Staphylococcus*				
Imipenem	12	0.186	0.064	0.25	0.032	4	100	0	0
Moxifloxacin	12	1.25	0.094	1.5	0.094	8	41.7	25	33.3
Gatifloxacin	12	1.75	0.125	2	0.094	8	41.7	8.3	50
Linezolid	12	1.75	2	2	0.75	4	100	0	0
Tigecycline	12	0.38	0.38	0.38	0.047	4	83.3	0	16.7
					*Pseudomonas aeruginosa*				
Imipenem	5	3	3	3	1.5	4	80	20	0
Moxifloxacin	5	3	4	3	1.5	4	nd	nd	nd
Gatifloxacin	5	2	4	2	0.75	4	60	40	0
Linezolid	5	>256	>256	>256	>256	>256	0	0	100
Tigecycline	5	>256	>256	>256	>256	>256	0	0	100
					Coagulase-Negative *Staphylococcus*				
Imipenem	4	0.064	0.064	0.064	0.012	0.125	100	0	0
Moxifloxacin	4	0.110	0.125	0.125	0.064	0.125	100	0	0
Gatifloxacin	4	0.156	N/A	0.19	0.064	0.25	100	0	0
Linezolid	4	3	4	4	0.15	4	100	0	0
Tigecycline	4	0.110	N/A	0.125	0.064	1	75	0	25
					Methicillin resistant *Staphylococcus Aureus*				
Imipenem	4	0.127	0.064	0.19	0.064	0.5	100	0	0
Moxifloxacin	4	0.139	0.047	0.23	0.047	0.25	100	0	0
Gatifloxacin	4	0.253	0.38	0.38	0.125	0.38	100	0	0
Linezolid	4	1.75	1.5	2	1.5	4	100	0	0
Tigecycline	4	0.25	0.25	0.25	0.19	0.75	80	0	20
					Alpha hemolytic *Streptococcus*				
Imipenem	3	3	3	3.0	0.064	3.0	33.3	0	66.6
Moxifloxacin	3	0.25	N/A	0.25	0.016	0.75	nd	nd	nd
Gatifloxacin	3	0.38	N/A	0.38	0.125	1.5	100	0	0
Linezolid	3	1.25	N/A	1.25	0.5	>256	66.6	0	33.3
Tigecycline	3	0.064	0.064	0.064	0.064	0.38	66.6	0	33.3
					*Bacillus Cereus*				
Imipenem	3	0.094	N/A	0.094	0.047	0.125	nd	nd	nd
Moxifloxacin	3	0.125	N/A	0.125	0.064	0.19	nd	nd	nd
Gatifloxacin	3	0.125	N/A	0.125	0.064	0.19	nd	nd	nd
Linezolid	3	3	N/A	3	1	4	nd	nd	nd
Tigecycline	3	0.125	N/A	0.125	0.047	0.75	nd	nd	nd

## Discussion

Etest® is a method of establishing antimicrobial susceptibility that, like disk diffusion (Kirby–Bauer test), is technically simple, but unlike the latter it produces semi-quantitative scores in micrograms per milliliter (μg/ ml). It consists of a thin plastic strip impregnated with antibiotic in a defined gradient, which is positioned on the agar plate surface (**Fig. 1**). The results are generally analogous with the quantitative outcomes of the standard MIC tests by broth microdilution or agar dilution [**5**,**10**].

The interpretation criteria for susceptibility tests are established on the in vitro behavior of a germ faced to an antimicrobial concentration akin to the achieved blood levels following enteral or parenteral administration. The cut-off points and their interpretation are generated considering many different criteria (microbiological, pharmacokinetical, pharmacodynamical and clinical). In other words, a pathogen is considered susceptible when it is inhibited by serum concentrations reached by the given antimicrobial at the recommended systemic dose [**7**,**10**]. These types of standards are established by microbiology research organizations, such as the Clinical & Laboratory Standards Institute (CLSI) or by committees expressly constituted, such as the European Committee on Antimicrobial Susceptibility Testing (EUCAST). Unfortunately, there are no internationally defined standards for all microorganisms [**8**,**9**].

Because there is no data on clinical trial results in humans, to be taken as guides for the treatment of patients with ophthalmic infections by means of the topical route, and since it is extremely unlikely that these head-to-head comparative clinical trials are ever to be executed due to costs, patient recruitment difficulties and marketing considerations, the mentioned in vitro susceptibility data taken from enteral or parenteral administration have been tools used for these purposes. However, it has been advocated that they should be examined with a group of descriptive statistical parameters in conjunction with a non-parametric statistical analysis without solely trusting one single parameter or test in all cases, to determine the in vitro superiority of one antimicrobial agent over another [**4**].

Following the mentioned existing standards, we established that in Gram-positive germs, the in vitro susceptibility values in descending order of the tested antimicrobials were: imipenem 90.3%, linezolid 87.9%, tigecycline 78.1%, gatifloxacin 68.8% and moxifloxacin 68.8%. However, some researchers have suggested that since there is not supportive data for intermediate susceptibility when analyzing ocular isolates, and due to the high antibiotic concentrations reached in the ocular tissues using a topical route of delivery, intermediate values should be interpreted as susceptible [**11**]. Adopting this concept for the analysis in the Gram-positive microorganisms, the in vitro susceptibility (now including also intermediate susceptibility) for both fourth-generation quinolones (gatifloxacin and moxifloxacin) increased to 75%. According to our in vitro antibiotic susceptibility analysis, imipenem and linezolid seemed to be the best options for all the Gram-positive microorganisms, since in general, they showed the highest susceptibility data. 

The in vitro values for Gram-negative susceptibility, in descending order, were: imipenem 72.7%, gatifloxacin 70%, moxifloxacin 66.7%, tigecycline 22.2%, and linezolid 0%. It is important to note, however, that for moxifloxacin we only located reference cut-off points for three of the pathogens, and they were not located for P. aeruginosa, so this susceptibility data cannot be considered. In addition, being linezolid active on Gram positive but not on Gram negative bacteria, 0% was an expected result of susceptibility among this latter group of microorganisms. According to Kowalski, considering the intermediate susceptibility values as susceptible, the proportion of susceptible isolates for imipenem and gatifloxacin increased to 90.9% and 90%, respectively [**11**].

The antibiotic with the highest susceptibility among Gram negatives was imipenem, followed by gatifloxacin and moxifloxacin. 

Imipenem, linezolid and tigecycline were incorporated inside the antimicrobials tested in our study as potential new alternatives in the topical treatment of ophthalmic infections. Imipenem is classified in the group of carbapenems, beta-lactam antibiotics that are highly resistant to beta-lactamase. Like all other antimicrobials of this family, imipenem inhibits cell wall synthesis by binding to and deactivating particular enzymes necessary for cross-linkage of bacterial cell wall peptidoglycan (transpeptidases that bind to penicillin and other antibiotics of the β-lactam class and, therefore, are known as penicillin-binding proteins) [**12**].

Linezolid is included in the class of compounds called oxazolidinones and acts by inhibiting the initiation phase of synthesis of proteins by combining with rRNA on both the 30S and 50S subunits and averting the creation of the 70S initiation complex in the ribosome, which can decrease the dimension of peptide chains and diminish the rate of translation. It may also preclude the appearance of virulence elements, leading to a decrease of toxins produced by Gram-positive germs, but, as previously commented, has no activity against Gram-negative bacteria [**13**].

Topical use of both imipenem and linezolid has been reported in few cases of corneal infections [**14**-**21**]. Furthermore, Elsahn et al. reported that 21 isolates of Staphylococcus aureus and 29 of methicillin-resistant coagulase-negative Staphylococcus recovered from infectious keratitis cases showed 100% in vitro susceptibility for linezolid [**22**].

Tigecycline, the first in the class of the glycylcycline antibiotics, is a derivative of minocycline. It can be classified as a third-generation tetracycline, which inhibits synthesis of proteins by blocking the binding of aminoacyl-tRNA to the ribosomal acceptor site. It is active against most Gram-positive, Gram-negative, anaerobic, atypical, and multidrug-resistant organisms, but has reduced activity against Pseudomonas spp., which was also observed in the present study (100% resistance) [**23**]. There are no reports regarding the use of topical tigecycline in ophthalmology. Though, recent experimental studies in animal models indicate that tigecycline could be a conceivable candidate as an ocular topical antibiotic. Goktas et al. studied the efficacy of tigecycline (10 or 50 mg/ mL topically applied) compared to vancomycin (50 mg/ mL) and saline solution in cases of experimental keratitis in rabbits, caused by local injection of methicillin-resistant Staphylococcus aureus (MRSA) bacteria. The researchers found that both treatment options significantly lower post intervention CFUs, with no statistically significant differences between them [**24**]. Romanowski et al., also studied topical 0.5% tigecycline in vitro activity and in vivo tolerability and efficacy in rabbit models with induced MRSA keratitis, compared to vancomycin 5% and saline; tigecycline showed lower MICs than against MRSA and other gram-positive bacteria, while MICs against gram-negatives (including Pseudomonas aeruginosa) were in treatable ranges with aggressive topical therapy. Tigecycline and vancomycin demonstrated similar effectiveness, but the first one showed no corneal adverse effects [**25**].

The growing resistance of the fluoroquinolones of the fourth generation, is a reality. A study published a few years ago (2016) reported that for coagulase-negative Staphylococcus, which caused endophthalmitis, the percentage of resistance to moxifloxacin in a United States institution increased from 21% in the period 1995–1999 to 62% in the period 2010–2014 [**26**]. The fourth-generation fluoroquinolone resistance in our series (samples collected in 2014) did not reach such worrying levels. Nevertheless, 25% of 32 Gram-positive isolates resulted resistant to gatifloxacin and 17.9% of 28 Gram-positive isolates resistant to moxifloxacin. In a previous study in our institution, with specimens from cases of keratitis and intraocular infections in the period 2011-2012, and in which susceptibility was evaluated with the Kirby-Bauer agar diffusion method, we found that 0% and 1.1% of the isolated Gram positives were resistant to moxifloxacin and gatifloxacin, respectively. Regarding Gram-negatives, the percentage of gatifloxacin resistance also increased compared to our previous study: 11.1% versus 0%. , In the present study, we were unable to determine the susceptibility profile of Gram-negatives to moxifloxacin because we did not find current accepted standards of the MIC cut-off point. The increased data found in this new series could be an indicator of a growing resistance to these agents in our environment.

Comparing the findings for imipenem in the current study (resistance of 9.7% for Gram-positives and 10% for Gram-negatives), it also showed an upsurge in its resistance rate, in comparison with the research conducted in our clinic with samples obtained between 2011 and 2012: resistance to imipenem with the Kirby-Bauer method was 1.1% among Gram-positive bacteria and 0% in Gram-Negatives [**27**].

As already commented, there are no CIM cut-off points established for antimicrobials when used topically, which makes it problematic to interpret their MIC based on serum concentrations. Nonetheless, previous researches have shown a parallel among the MIC value and clinical outcomes in cases topically treated with those antibiotics. Thus, it is deemed suitable to establish the MIC of antimicrobial ophthalmic eye drops as a guide to treat ocular infections [**28**-**30**].

Sueke and co-authors analyzed 772 bacteria cultures collected from keratitis cases in the United Kingdom between 2003 and 2006. They reported that all microorganisms, with the exception of P. Aeruginosa, were susceptible to third-generation tetracycline (tigecycline). They also studied meropenem and linezolid, and established that, due to its broad spectrum, meropenem could be a suitable alternate for early empirical monotherapy in bacterial keratitis [**31**]. In addition, tigecycline and linezolid could be options to cover Gram-positives and used together with an antimicrobial that covers Gram-negatives for a broader coverage [**12**,**13**,**23**].

In the analyzed group of microorganisms in the present study, the susceptibility percentage of imipenem and linezolid antimicrobials against Gram-positive microorganisms were above 87%, and for tigecycline this value was 78.1%. In Gram negatives, imipenem reached a susceptibility percentage of 70%. Differently, the susceptibility values for gatifloxacin and moxifloxacin in Gram-positive microorganisms (even including the intermediate susceptibility values as susceptible), were slightly lower: 75%.

The results of the present study led us to the conclusion that, although fourth generation fluoroquinolones (gatifloxacin and moxifloxacin) are presently employed as initial empirical monotherapy in our clinic, given the rising resistance to them, any of the new antimicrobials in ophthalmology we studied (imipenem, linezolid or tigecycline) could be employed as an alternate treatment in case of finding Gram-positive germs in the initial staining. If Gram-negatives are initially identified, due to the lack of an antimicrobial with a susceptibility percentage above 80%, an assessment regarding any of the synergies proposed by some groups of researchers (or any antibiotic group similar to those studied) could be used. Sueke and co-authors suggested the use of meropenem in conjunction with ciprofloxacin [**31**]. A similar combination that could be evaluated is a fourth-generation quinolone with imipenem. On the other hand, Suzuki and co-authors suggested the combination of gatifloxacin and cefmenoxime [**32**]. 

Since the bacterial ophthalmic infections (both corneal, keratitis, and intraocular, endophthalmitis) are still one very important cause of eye disease, unquestionably, it is critical to improve the clinical results with antimicrobial agents [**33**]. Further studies on this topic are warranted.

**Acknowledgments**

We would like to thank Fundación Oftalmológica de Santander FOSCAL, the Higuera Escalante-OCULAB Clinical Laboratory and Universidad Autónoma de Bucaramanga UNAB.

**Declaration of interests**

None of the authors has any interest in any of the equipment or substances mentioned in this article, which could generate a conflict.

**Financial disclosures**

The Universidad Autónoma de Bucaramanga UNAB financed the study, through a contribution assigned in the VII INTERNAL CONVOCATION FOR RESEARCH PROJECTS UNAB 2013-2014. Proposal code C34056.
